# Structural equation modeling (SEM) of kidney function markers and longitudinal CVD risk assessment

**DOI:** 10.1371/journal.pone.0280600

**Published:** 2023-04-20

**Authors:** Ryosuke Fujii, Roberto Melotti, Martin Gögele, Laura Barin, Dariush Ghasemi-Semeskandeh, Giulia Barbieri, Peter P. Pramstaller, Cristian Pattaro

**Affiliations:** 1 Institute for Biomedicine (Affiliated to the University of Lübeck), Eurac Research, Bolzano, Bozen, Italy; 2 Department of Preventive Medical Science, Fujita Health University School of Medical Sciences, Toyoake, Japan; 3 Department of Human Genetics, Leiden University Medical Center, Leiden, The Netherlands; 4 Department of Neurosciences, Biomedicine and Movement Sciences, University of Verona, Verona, Italy; The University of the West Indies, JAMAICA

## Abstract

Lower kidney function is known to enhance cardiovascular disease (CVD) risk. It is unclear which estimated glomerular filtration rate (eGFR) equation best predict an increased CVD risk and if prediction can be improved by integration of multiple kidney function markers. We performed structural equation modeling (SEM) of kidney markers and compared the performance of the resulting pooled indexes with established eGFR equations to predict CVD risk in a 10-year longitudinal population-based design. We split the study sample into a set of participants with only baseline data (n = 647; model-building set) and a set with longitudinal data (n = 670; longitudinal set). In the model-building set, we fitted five SEM models based on serum creatinine or creatinine-based eGFR (eGFRcre), cystatin C or cystatin-based eGFR (eGFRcys), uric acid (UA), and blood urea nitrogen (BUN). In the longitudinal set, 10-year incident CVD risk was defined as a Framingham risk score (FRS)>5% and a pooled cohort equation (PCE)>5%. Predictive performances of the different kidney function indexes were compared using the C-statistic and the DeLong test. In the longitudinal set, a SEM-based estimate of latent kidney function based on eGFRcre, eGFRcys, UA, and BUN showed better prediction performance for both FRS>5% (C-statistic: 0.70; 95% CI: 0.65–0.74) and PCE>5% (C-statistic: 0.75; 95%CI: 0.71–0.79) than other SEM models and different eGFR formulas (DeLong test *p*-values<3.21×10^−6^ for FRS>5% and <1.49×10^−9^ for PCE>5%, respectively). However, the new derived marker could not outperform eGFRcys (DeLong test *p*-values = 0.88 for FRS>5% and 0.20 for PCE>5%, respectively). SEM is a promising approach to identify latent kidney function signatures. However, for incident CVD risk prediction, eGFRcys could still be preferrable given its simpler derivation.

## Introduction

Chronic kidney disease (CKD) is an age-related pathophysiological condition affecting ~840 million individuals worldwide [1] and predicted to become the fifth global cause of death by 2040 [2]. In addition to increasing the risk of end-stage kidney disease, CKD and kidney dysfunction are known to enhance cardiovascular disease (CVD) risk [3–7].

Kidney function is assessed via the glomerular filtration rate (GFR). Given the impossibility to measure the true GFR, population-based studies usually estimate it through endogenous markers such as serum creatinine (SCr) or cystatin C (Cys) or both [8–12]. Additional informative markers include blood urea nitrogen (BUN), uric acid (UA), and serum albumin (Alb) [13]. However, none of these markers is the exclusive reflection of kidney function, each one being influenced by other metabolic pathways and homeostatic conditions [14]. SCr depends on age, sex, muscle mass and food intake [15]. BUN and UA depend on liver metabolism and endocrine function [16]. Cys may reflect inflammation [17] and thyroid hormone metabolism [18]. eGFRcre and eGFRcys are not always consistent with each other and they may differ in terms of CKD classification [19, 20]. Combining SCr and Cys together usually improves estimate of the true kidney function level [11, 12].

While kidney dysfunction does increase CVD risk. Recently, a combination of ten non-routinely measured urine and blood kidney biomarkers has been shown to improve CVD risk prediction [21]. However, it is unclear whether and to which extent there is an overlap between the different standard kidney function markers in predicting CVD risk. Particularly unexplored is the possibility to integrate kidney function markers into a structural equation modeling (SEM) framework. This technique was widely applied to social and behavioral sciences to identify non-observable latent traits undergoing observable psychological or psychiatric manifestations [22, 23]. Only more recently, SEM has been applied to biomarker research [24]. If we consider each kidney-related marker as a partial manifestation of the true underlying kidney function level, we can integrate all of them into a SEM framework and obtain an estimate of the kidney function level as a latent unobserved trait.

The aim of our analysis was to assess whether the combination of routinely used kidney function markers (SCr, Cys, BUN, and UA) into a SEM framework could improve the prediction of CVD risk over the individual markers. To answer this question, we exploited data from a population-based study with a 10-year follow-up.

## Materials and methods

### Study design

**Fig 1:**
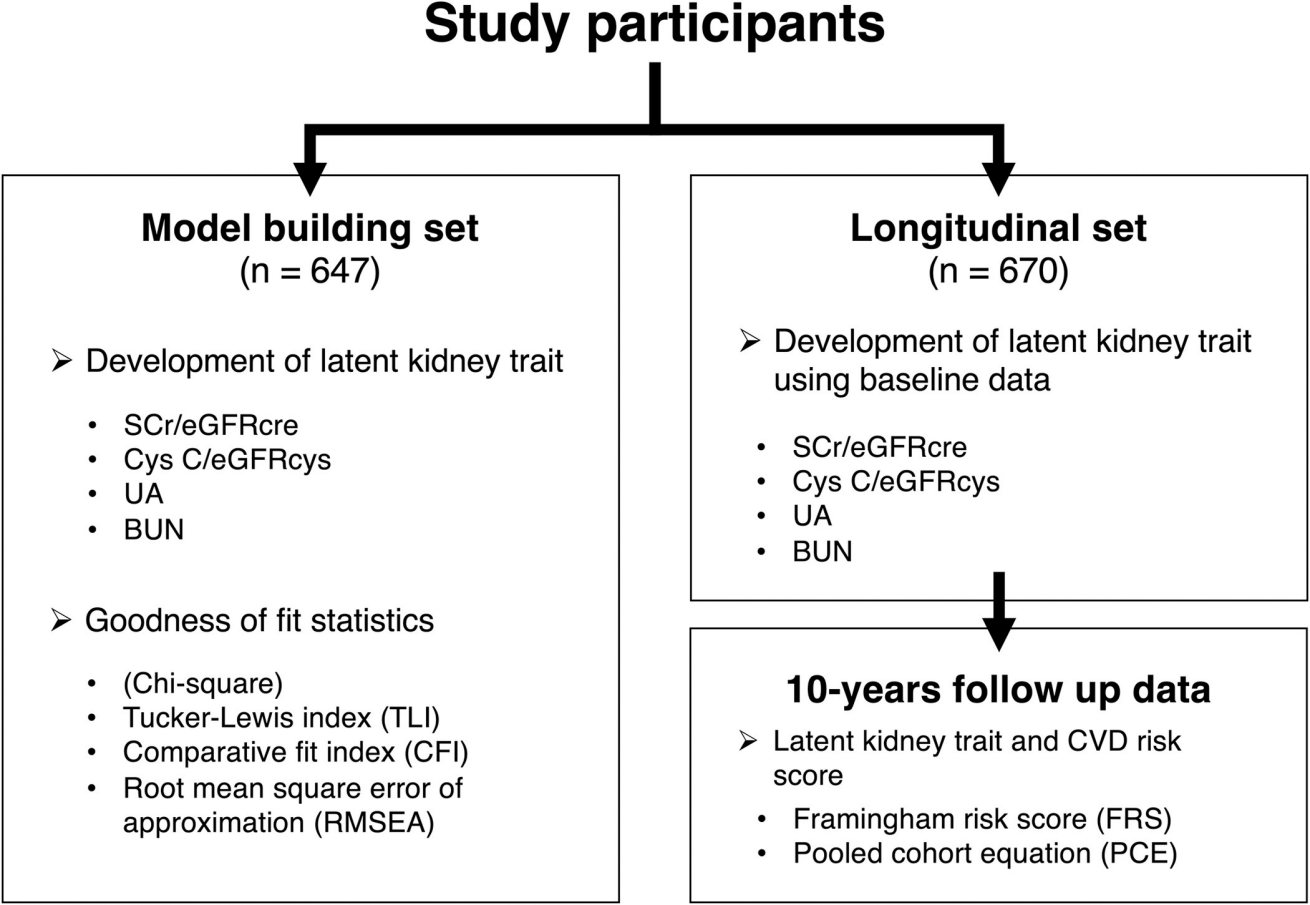
Analysis flowchart. All study participants are divided into two parts: Model building set (n = 647, left panel) and Longitudinal set (n = 670, right panel).

This work was based on the Microisolates in South Tyrol (MICROS) study, a cross-sectional population-based study on 1,357 adults conducted in South Tyrol, Italy, in 2002 and 2003 [25, 26]. The study participants are mainly recruited in the following villages: Vallelunga/Langtaufers, Martello/Martell, and Stelvio/Stilfs. Of these participants, 733 participated also to the Cooperative Health Research in South Tyrol (CHRIS) study, an ongoing prospective study with similar protocol and insisting on same geographical district, which recruited participants between 2011 and 2018 [27]. We thus split the MICROS baseline sample into a cross-sectional set, where only baseline data were available (*n* = 647), and a longitudinal set with ~10-year follow-up (*n* = 670). We used the cross-sectional set for model development and the longitudinal set to assess the ability of kidney function estimators developed in the cross-sectional set to predict incident CVD risk over 10 years. See flowchart in Fig 1. The CHRIS study was approved by the Ethics Committee of the Healthcare System of the Autonomous Province of Bolzano (Südtiroler Sanitätsbetrieb/Azienda Sanitaria dell’Alto Adige), protocol no. 21/2011 (19 Apr 2011). Within their framework, our project has been approved by the Access Committee for Data and Sample collections of the Institute for Biomedicine (No.344). All participants gave written informed consent.

### Data collection and laboratory analyses

In the MICROS study (baseline), participants’ demographic and clinical history were collected by interviewers using standardized questionnaires. Blood samples were collected after overnight fasting. Samples underwent routine biochemical analyses at the local hospital. Serum aliquots were stored at -80°C until subsequent measurement. Serum levels of SCr, Cys, UA, BUN, and Alb were measured at the Institute for Clinical Chemistry and Laboratory Medicine, Regensburg University Medical Center, Germany, as previously described [19].

In the CHRIS study (follow-up), routine biochemical measurements were performed at the Meran/Merano hospital as described previously [28]. Relevant to this study are total cholesterol (TC) and high-density lipoprotein cholesterol (HDL-C). Considered here are also information on antihypertensive therapy, history of diabetes, and smoking habits [29] collected through computer-assisted interviewer-administered questionnaires, and blood pressure measured on site in supine position after 20 minutes resting. Questionnaire-based variables for both MICROS and CHRIS, including questions, answer options and coding, are summarized in S1 Table.

### GFR estimation in the MICROS study

GFR was estimated with: the Modification of Diet in Renal Disease (MDRD) study equations with 4 (eGFR_MDRD4_) [8] and 6 parameters (eGFR_MDRD6_) [9]; the 2009 SCr-based Chronic Kidney Disease Epidemiology Collaboration (CKD-EPI) formula (eGFR_CKD-EPI Cre 2009_) [10]; the Cys-based CKD-EPI formula (eGFR_CKD-EPI Cys_) [11]; the new race-free CKD-EPI formula with both Cre and Cys (eGFR_CKD-EPI CreCys_) [12]; and the new race-free SCr-based CKD-EPI formula (eGFR_CKD-EPI Cre 2021_) [12]. We used the R package “*nephro*” (ver.1.3) (https://cran.r-project.org/web/packages/nephro/index.html) [19]. Details are shown in S2 Table.

### Outcome definition at follow-up

In the CHRIS study, we estimated the Framingham risk score (FRS) [30] and the pooled cohort equation (PCE) score [31]. The FRS estimates the risk of any CVD event, while the PCE is focused on the risk of hard atherosclerotic CVD. Both risk scores consist of conventional CVD risk factors (age, sex, TC, HDL-C, systolic blood pressure, antihypertensive therapy, history of diabetes, and current smoking). Additionally, PCE includes a race term (irrelevant for our study which was based exclusively on European-ancestry individuals). Based on cutoffs proposed by the American College of Cardiology and the American Heart Association, we dichotomized both FRS and PCE risks as “low risk” (score<5%) or “risk” (>5%) [32].

### Statistical analysis

**Fig 2:**
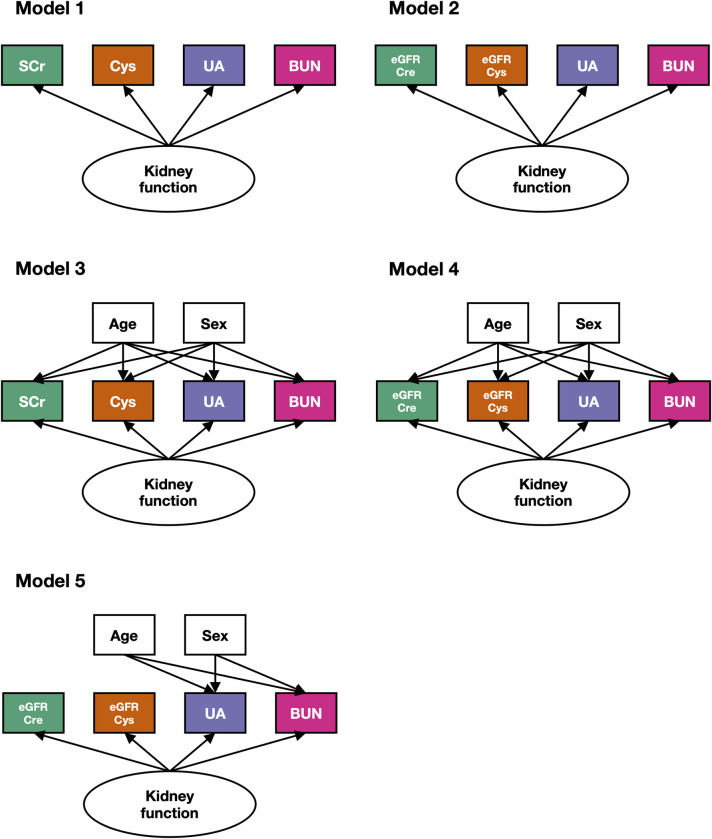
SEM conceptual framework under different assumptions (model 1–5). We developed five models based on different combinations of kidney biomarkers and demographic variables (sex and age). Circles represent latent variables and squares represent observed variable. BUN: blood urea nitrogen; Cys: cystatin C; eGFRcre: creatinine-based eGFR (eGFR_CKD-EPI Cre 2021_); eGFRcys: cystatin c-based eGFR (eGFR_CKD-EPI Cys_); SCr: serum creatinine; UA: uric acid.

In the model-building set, we estimated a latent kidney trait from five different SEM models (Fig 2): model 1 included the simple biomarkers SCr, Cys, UA, and BUN; model 2 included eGFRcre (eGFR_CKD-EPI Cre 2021_), eGFRcys (eGFR_CKD-EPI Cys_), UA, and BUN; model 3 additionally accounted for sex and age for each variable in model 1; model 4 was like model 3, but replacing SCr and Cys with eGFRcre and eGFRcys; model 5 was a reduced form of model 4, incorporating age and sex only for UA and BUN. Goodness of fit was assessed with the confirmatory factor index (CFI) and the root mean square error of approximation (RMSEA) [33]. In the longitudinal set, we applied logistic regression analyses to compare the predictive ability for 10-year CVD risk among ten kidney-related variables (four SEM-based kidney traits and six eGFR formulas). We created a receiver operating characteristics (ROC) curves to assess the markers’ predictive performance based on the C-statistics. To test statistical difference of C-statistics between two kidney indices, we also performed the DeLong test. As an additional analysis for continuous outcomes (lopg-transformed FRS and PCE), we also performed linear regression analyses to estimate an explained variance of CVD risk scores by the kidney traits. All statistical analyses were performed using the statistical software R ver.4.0.0 (http://www.R-project.org). The R packages of lavaan (ver.0.6–11) [34] is used for SEM analysis and pROC (ver.1.18.0) [35] is for drawing ROC curves and performing the DeLong test, respectively.

## Results

### Characteristics of the model-building and longitudinal dataset

Table 1 summarizes the demographic and clinical characteristics of study participants included in the model-building and longitudinal datasets, respectively. The proportion of females was similar across the two sets: 57.8% in the model-building set and 55.3% in the longitudinal set. Participants were older in the model-building as compared to the longitudinal set (mean age: 49.4 versus 40.2 years, respectively). Accordingly, the model-building set had higher prevalence of diabetes and hypertension. Interestingly, whether the eGFR was higher in the model-building or in the longitudinal-set depended on the GFR estimating equation.

**Table 1:** Baseline characteristics of participants included in the model-building and the longitudinal sets^a^.

Variables	Model-building set (n = 647)	Longitudinal set (n = 670)
Women	374 (57.8%)	371 (55.3%)
Age, years	49.4 (19.1)	40.2 (13.5)
Serum creatinine, mg/dl	0.86 (0.17)	0.85 (0.15)
Uric acid, mg/dl	5.46 (1.63)	5.18 (1.31)
Blood urea nitrogen, mg/dl	17.5 (5.1)	16.3 (4.1)
Cystatin C, mg/l	0.83 (0.25)	0.75 (0.13)
Serum albumin, g/dl	4.72 (3.4)	4.76 (3.3)
eGFR_CKD-EPI Cre 2009_, ml/min/1.73m^2^	105.9 (23.2)	97.6 (16.9)
eGFR_CKD-EPI Cre 2021_, ml/min/1.73m^2^	95.0 (19.4)	100.8 (16.1)
eGFR_CKD-EPI Cys_, ml/min/1.73m^2^	100.7 (24.5)	111.3 (15.7)
eGFR_CKD-EPI CreCys_, ml/min/1.73m^2^	101.4 (21.3)	109.5 (14.2)
eGFR_MDRD4_, ml/min/1.73m^2^	102.8 (24.1)	88.8 (21.9)
eGFR_MDRD6_, ml/min/1.73m^2^	102.4 (23.1)	91.2 (19.9)
10-year FRS>5%^b^^,d^	–	326 (60.9%)
10-year PCE>5%^c,d^	–	216 (40.4%)
Diabetes	30 (5.3%)	8 (1.3%)
Hypertension	133 (23.0%)	69 (11.0%)

^a^Quantitative variables are presented as mean (standard deviation), while categorical variables are shown as n (%).

^b^These analyses were performed in 535 participants without previous CVD clinical history.

CKD-EPI: the Chronic Kidney Disease Epidemiology Collaboration; Cre: creatinine; Cys: cystatin C; FRS: Framingham risk score; MDRD: the Modification of Diet in Renal Disease study; PCE: pooled cohort equation

### The model-building set

The standardized factor loadings estimated under the different structural equation models are summarized in Table 2. In general, SCr or eGFRcre (eGFR_CKD-EPI Cre 2021_) obtained the highest loading (>0.75 in models 1, 2, and 5), followed by Cys or eGFRcys (eGFR_CKD-EPI Cys_). In the five models, loadings displayed different patterns: in model 1, SCr received a much larger weight than Cys, UA and BUN, which had all similar loadings. Models 2 and 5 gave more weight to eGFRcre and eGFRcys, compared with UA and BUN. In addition, model 2 gave more weight to BUN than UA, in contrast to model 5. In model 3, the largest loading was assigned to SCr, followed by Cys, UA, and BUN. In Model 4, the loadings magnitude was similar for eGFRcre, eGFRcys and UA, while it was lowest for BUN. In terms of goodness of fit (Table 2), all models, except model 5, showed a CFI higher than 0.95 indicating good fitting. The RMSEA indicated excellent fit for model 3 (RMSEA = 0.024), borderline levels for models 1, 2, and 4, and extremely poor fit for model 5. Model 5 was not considered any further for CVD prediction analysis.

**Table 2:** Factor loadings and goodness-of-fit statistics for the five SEM models.

Items	Model 1	Model 2	Model 3	Model 4	Model 5
*Standardized factor loadings* ^*a*^					
SCr/eGFRcre	0.757	0.889	0.621	0.432	0.863
Cys/eGFRcys	0.597	0.856	0.541	0.415	0.886
UA	0.597	-0.393	0.362	-0.309	-0.483
BUN	0.638	-0.561	0.415	-0.441	-0.577
*Goodness-of-fit statistics*					
CFI	0.978	0.977	0.999	0.989	0.612
RMSEA	0.099	0.126	0.024	0.106	0.436

^a^Factor loadings are all direction-concordant in Models 1 and 3, where SCr, Cys, BUN and UA were included, as all 4 markers have direction-concordant association with kidney function; in models 2, 4, and 5, we included eGFRcre and eGFRcys, which are associated with function in the opposite direction as compared to BUN and UA.

BUN: blood urea nitrogen; CFI: confirmatory factor index; eGFRcre: creatinine-based eGFR (eGFR_CKD-EPI Cre 2021_); eGFRcys: cystatin C-based eGFR (eGFR_CKD-EPI Cys_); RMSEA: root mean square error of approximation; SCr: serum creatinine; SEM: structural equation model; UA: uric acid.

### Distribution of the estimated latent kidney traits and their relation with eGFR equations

**Fig 3:**
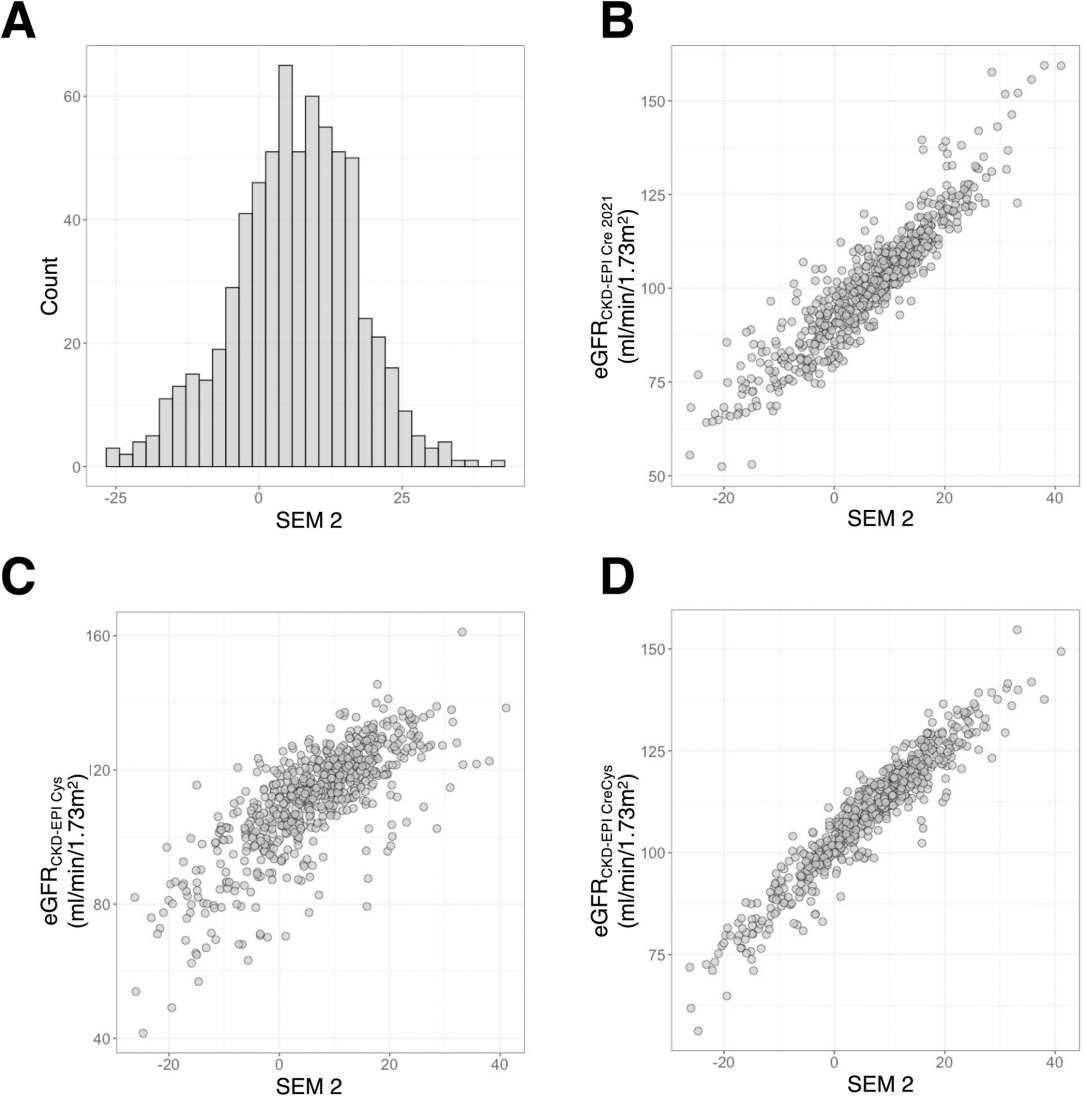
The distribution of latent kidney trait from model 2 (SEM 2). **Panel A**: Histogram of SEM 2. **Panel B**: Scatter plot for SEM 2 and eGFR_CKD-EPI Cre 2021_. **Panel C**: Scatter plot for SEM 2 and eGFR_CKD-EPI Cys_. **Panel D**: Scatter plot for SEM 2 and eGFR_CKD-EPI CreCys_.

The latent kidney trait estimated from model 2 (SEM 2) in the longitudinal set was normally distributed (Fig 3A). The latent traits estimated with models 1, 3, and 4 were also nearly normal (S1 Fig). SEM 2 was highly correlated with eGFR_CKD-EPI Cre 2021_ (Pearson’s correlation coefficient *r* = 0.92; Fig 3B), eGFR_CKD-EPI Cys_ (*r* = 0.75; Fig 3C) and eGFR_CKD-EPI CreCys_ (*r* = 0.95; Fig 3D). The positive correlation with eGFR estimates implies that lower SEM 2 indicates lower kidney function. Differently from SEM 2, SEM 1 was poorly correlated with the eGFR estimates and did not capture well the sex stratification (S2 Fig). While accommodating the sex stratification of kidney function better than SEM 1, SEM 3 and 4 still showed limited correlation with eGFR estimates (S3 and S4 Figs).

### Prediction analysis

**Fig 4:**
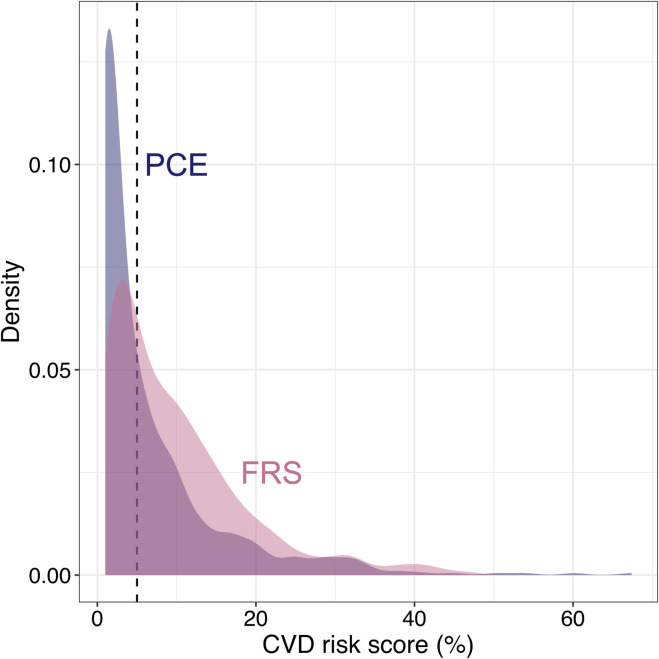
Distribution of Framingham risk score (FRS) and pooled cohort equation (PCE) in longitudinal set. Pink- and blue-colored density plots corresponds to FRS and PCE, respectively. The grey dotted line indicates the cut-off value for dichotomization (5%).

**Fig 5:**
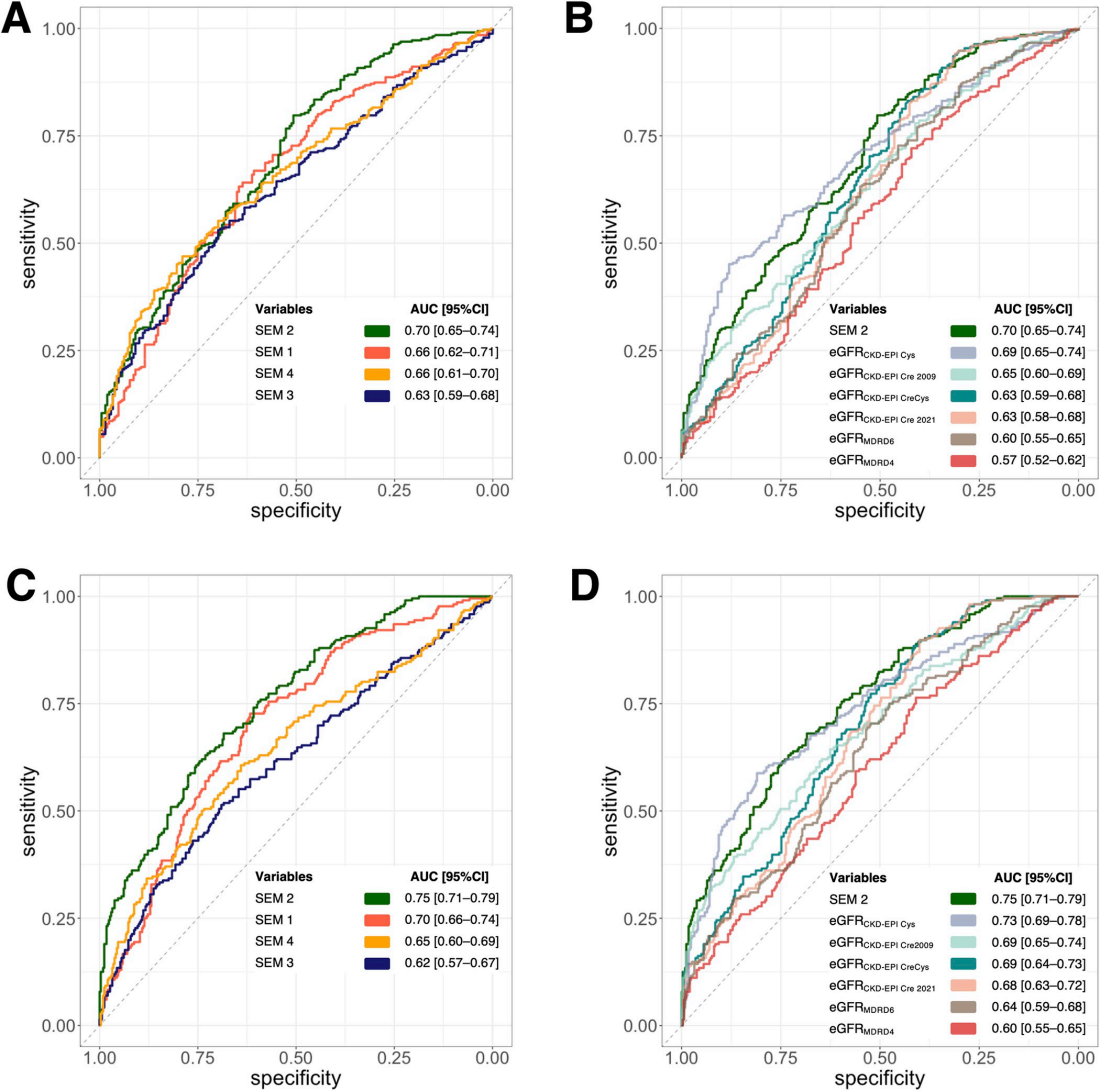
Comparisons of receiver operating characteristics (ROC) curves for Framingham risk score (FRS)>5 for general CVD risk estimation and pooled cohort equation (PCE)>5 for risk estimation of hard atherosclerotic cardiovascular disease in 10 years. **Panel A**: Comparisons of the C-statistics for FRS>5 within SEM-based latent kidney traits. **Panel B**: Comparisons of the C-statistics for FRS>5 with the 2^nd^ SEM model (SEM 2) and eGFR equations. **Panel C**: Comparisons of the C-statistics for PCE>5 within SEM-based latent kidney traits. **Panel D**: Comparisons of the C-statistics for PCE>5 with the 2^nd^ SEM model (SEM 2) and eGFR equations. The x-axis shows specificity ranging from 1 to 0, while the y-axis shows sensitivity ranging from 0 to 1. The right-hand table summarizes the C-statistics and its 95% confidence intervals in descending order. CKD-EPI: the Chronic Kidney Disease Epidemiology Collaboration; Cre: creatinine; Cys: cystatin C; eGFR: estimated glomerular filtration rate; MDRD: the Modification of Diet in Renal Disease study; SEM: structural equation modeling.

Overall, 326 (60.9%) individuals were classified at high CVD risk with the FRS, and 216 (38.7%) with the PCE (Fig 4). The second SEM-derived index, SEM 2, showed better performance (C-statistic: 0.70; 95% confidence interval, CI: 0.65–0.74) than all other SEM-derived indexes to predict FRS>5% over 10 years, even if its advantage was not uniform across the entire sensitivity and specificity spectrum (Fig 5A). SEM 2 AUC was not significantly different from that of eGFR_CKD-EPI Cys_ (C-statistic: 0.69, 95%CI: 0.65–0.74; DeLong test *p*-value for comparison: 0.88; Fig 5B). However, SEM 2 showed better prediction properties than eGFR_CKD-EPI Cre 2009_ (C-statistic: 0.65; 95%CI: 0.60–0.69; *p* = 3.21×10^−6^), eGFR_CKD-EPI Cre 2021_ (C-statistic: 0.63; 95%CI: 0.58–0.68; *p* = 2.33×10^−9^), eGFR_CKD-EPI CreCys_ (C-statistic: 0.63; 95%CI: 0.59–0.68; *p* = 2.03×10^−14^), eGFR_MDRD4_ (C-statistic: 0.57; 95%CI: 0.52–0.62; *p*<2.2×10^−16^), and eGFR_MDRD6_ (C-statistic: 0.60; 95%CI: 0.55–0.65; *p* = 6.51×10^−14^).

For the prediction of a PCE of >5% over 10 years, SEM 2 showed the best predictive performance over all other SEM-based markers, uniformly across all sensitivity and specificity levels (C-statistic: 0.75; 95%CI: 0.71–0.79; Fig 5C). Similar to the FRS case, SEM 2 did not outperform the eGFR_CKD-EPI Cys_ (C-statistic: 0.73; 95%CI: 0.69–0.78; *p* = 0.20; Fig 5D), but it did perform better than the eGFR_CKD-EPI Cre 2009_ (C-statistic: 0.69; 95%CI: 0.65–0.74; *p* = 1.49×10^−9^), eGFR_CKD-EPI Cre 2021_ (C-statistic: 0.68, 95%CI: 0.63–0.72; *p* = 2.07×10^−13^), eGFR_CKD-EPI CreCys_ (C-statistic: 0.69; 95%CI: 0.64–0.73; *p*<2.2×10^−16^), eGFR_MDRD4_ (C-statistic: 0.60; 95%CI: 0.55–0.65; *p*<2.2×10^−16^), and eGFR_MDRD6_ (C-statistic: 0.64; 95%CI: 0.59–0.68; *p*<2.2×10^−16^).

To corroborate these findings, we also fitted linear regression models on the logarithm of the FRS and PCE score, to estimate the variance explained by each marker. The regression r-squared for the FRS was of 0.20 for SEM 2, 0.16 for eGFR_CKD-EPI Cys_, 0.11 for eGFR_CKD-EPI CreCys_, 0.11 for eGFR_CKD-EPI Cre 2009_, 0.09 for eGFR_CKD-EPI Cre 2021_, 0.06 for the eGFR_MDRD6_, and 0.03 for the eGFR_MDRD4_. For the PCE, we observed the following r-squared: 0.28 for SEM 2, 0.23 for eGFR_CKD-EPI Cys_, 0.18 for eGFR_CKD-EPI CreCys_, 0.16 for eGFR_CKD-EPI Cre 2009_, 0.14 for eGFR_CKD-EPI Cre 2021_, 0.08 for the eGFR_MDRD6_, and 0.05 for the eGFR_MDRD4_.

## Discussion

In a population-based study of European individuals, we applied SEM to four kidney markers. Our aim was to assess whether the integration of multiple markers could outperform standard kidney function estimates based on a single marker in terms of incident CVD risk prediction. By leveraging an independent longitudinal dataset, we found that a SEM-based kidney function index and eGFRcys performed better than all other commonly used GFR formulas, in terms of predicting 10-year incident CVD risk.

The motivation to fit a structural equation model came from previous studies that tried to combine multiple markers of kidney health. Lee AK et al. [21] reported that integration of various kidney biomarkers improved the prediction accuracy of CVD mortality compared with conventional kidney indices. As manifest variables, they used ten kidney biomarkers, including kidney injury molecule-1 (KIM-1) and uromodulin. Another experimental study estimated latent kidney function traits based on different biomarkers and examined the performance in animal model [36]. They used a two-factor model for latent variables, where the two factors represented kidney damage and kidney function, incorporating KIM-1 and other biomarkers. These studies used specific molecular markers of tubular injury and tubular reserve, which are not commonly measured in clinical practice. Our attempt was instead based on common markers that, together with basic demographic variables such as age and sex, are measured in most population-based studies. This would have broadened clinical utility of our results.

The best model from our analyses was SEM 2, which pooled together eGFRcre (eGFR_CKD-EPI Cre 2021_), eGFRcys (eGFR_CKD-EPI Cys 2012_), BUN, and UA, with factor loadings of 0.889, 0.856, -0.561, and -0.393, respectively. The factor loadings reflect the relation of the four markers with the true kidney function: positive and substantially equivalent for eGFRcre and eGFRcys, and negative and substantially smaller for BUN and UA. In terms of CVD risk prediction, taken as a binary trait, this model showed similar performance to using eGFRcys alone. When using linear modeling, SEM 2 explained a larger proportion of the FRS and the PCE than eGFRcys. It is worth highlighting that SEM 2 and eGFRcys outperformed eGFRcrecys in CVD risk prediction. A previous study reported that a combination of eGFRcre- and eGFRcys-based categories could improve prediction of CVD mortality in intensive care [37], but this may be a very different context compared to a general population situation, where most individuals are healthy or have a low burden of disease. The question remains as to why the performance of a single biomarker, eGFRcys, was not much inferior to SEM. A plausible reason might be that eGFRcys reflects both kidney function and components of the cardiovascular risk that are less dependent on kidney function. For instance, cystatin C levels are associated with obesity. A previous study has reported that eGFRcys reflects CVD risk better than eGFRcre [38]. This is in line with our results that show that eGFRcys outperformed all other eGFR estimates in terms of 10-year CVD risk prediction. The result that SEM 2 performed similar or slightly better than eGFRcys supports SEM 2 as a better solution than eGFR estimates not based on cystatin C. We believe that further explorations of SEM of kidney function should be attempted. In particular, two-factor modeling that separates the kidney function and the kidney damage aspects seems promising, especially in terms of CVD risk prediction. This study can be a first step towards more extensive research on multivariate approaches to kidney function modeling.

The main strength of our analysis was the presence of two independent sets, one used for model development and a second, longitudinal set used for model testing. Most studies have only performed either a search for the best-fitting SEM model or an association test between an outcome and a SEM-based index based on an arbitrary model [39–41]. In fact, our 3^rd^ SEM showed the best goodness of fit in the model building set, but this model did not show the best predictive ability in the longitudinal analysis. Combining results from the model building set (proving sufficient goodness of fit for SEM 2) and the longitudinal set (showing that SEM 2 was the best predictor), we followed a pragmatic approach focused on the purpose of our investigation. The fact that the two independent sets were derived from the same population in the same geographical region has probably provided further consistency across the two analyses.

The main limitation of our study was the lack of an objective GFR measurement to assess the performance of the latent trait estimation, although this limitation is common to most population-based studies. An additional limitation was the small sample size, implicating too few incidents cardiovascular events over the 10-year follow-up. Based on self-reported CKD and CVD events, in our study we observed an incidence of about 1%, which did not bear sufficient statistical power to assess the predictive performances of the fitted models. For this reason, we assessed the predictive ability of fitted models against the two CVD risk scores, the FRS and PCE score, which reflect pre-clinical conditions preceding CVD onset. Further studies that consider objective CVD events are warranted to confirm the significance of our approach. Finally, the generalizability and transportability of the estimated latent kidney function trait should be explored in different settings: our study participants were recruited from a specific geographical location in the Italian Alps, which might not be representative of different locations and demographic characteristics.

## Conclusion

Applying SEM to multiple, conventional kidney function markers is a promising approach to identify the underlying, unobserved true kidney function level. However, in an application that assessed the ability of kidney function markers to predict incident CVD risk over 10 years, SEM-based modeling was almost equivalent or just slightly better than eGFRcys, and both of them outperformed all other solutions. Given its simpler implementation over SEM, eGFRcys is probably still the best marker to assess the effect of kidney function on incident CVD risk.

## Supporting information

S1 Fig Distributions of latent kidney trait. **Panel A**: Distribution of estimated with the 1^st^ structural equation model (SEM 1). **Panel B**: Distribution of estimated with the 3^rd^ structural equation model (SEM 3). **Panel C**: Distribution of estimated with the 4^th^ structural equation model (SEM 4).
(PDF)

S2 Fig Scatter plots with latent kidney traits and eGFR_CKD-EPI Cre 2021_. **Panel A**: Scatter plot of estimated with the 1^st^ structural equation model (SEM 1) and eGFR_CKD-EPI Cre 2021_. **Panel B**: Scatter plot of estimated with the 3^rd^ structural equation model (SEM 3) and eGFR_CKD-EPI Cre 2021_. **Panel C**: Scatter plot of estimated with the 4^th^ structural equation model (SEM 4) and eGFR_CKD-EPI Cre 2021_.
(PDF)

S3 Fig Scatter plots with latent kidney traits and eGFR_CKD-EPI Cys_. **Panel A**: Scatter plot of estimated with the 1^st^ structural equation model (SEM 1) and eGFR_CKD-EPI Cys_. **Panel B**: Scatter plot of estimated with the 3^rd^ structural equation model (SEM 3) and eGFR_CKD-EPI Cys_. **Panel C**: Scatter plot of estimated with the 4^th^ structural equation model (SEM 4) and eGFR_CKD-EPI Cys_.
(PDF)

S4 Fig Scatter plots with latent kidney traits and eGFR_CKD-EPI CreCys_. **Panel A**: Scatter plot of estimated with the 1^st^ structural equation model (SEM 1) and eGFR_CKD-EPI CreCys_. **Panel B**: Scatter plot of estimated with the 3^rd^ structural equation model (SEM 3) and eGFR_CKD-EPI CreCys_. **Panel C**: Scatter plot of estimated with the 4^th^ structural equation model (SEM 4) and eGFR_CKD-EPI CreCys_.
(PDF)

S1 Table Questions, answer options, and coding for the questionaries in the MICROS and CHRIS study. (PDF)

S2 Table Different estimation formulas for GFR. (PDF)
