# Supplementary figures and images for: Structural equation modeling (SEM) of kidney function markers and longitudinal CVD risk assessment
Source: PLoS One. 2023 Apr 20;18(4):e0280600. doi: 10.1371/journal.pone.0280600 (PMC10118200; doi:10.1371/journal.pone.0280600)

**A**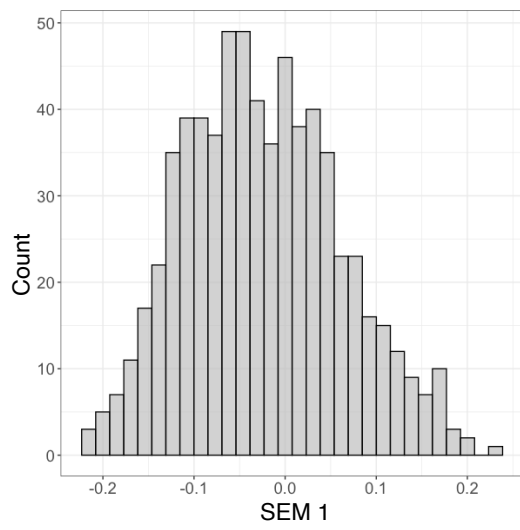**B**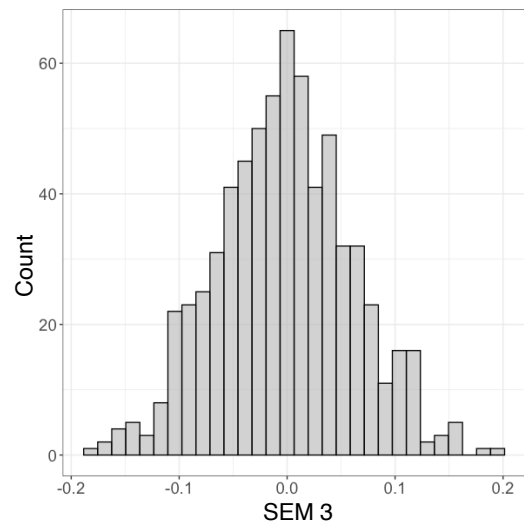**C**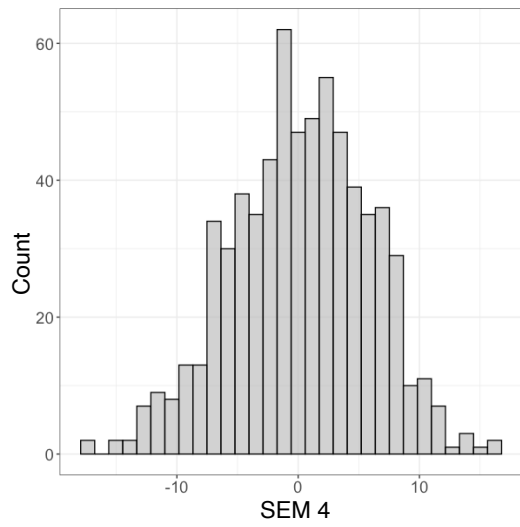

Supplement: S1 Fig — Panel A: Distribution of estimated with the 1st structural equation model (SEM 1). Panel B: Distribution of estimated with the 3rd structural equation model (SEM 3). Panel C: Distribution of estimated with the 4th structural equation model (SEM 4). (PDF) [file pone.0280600.s001.pdf]

**A**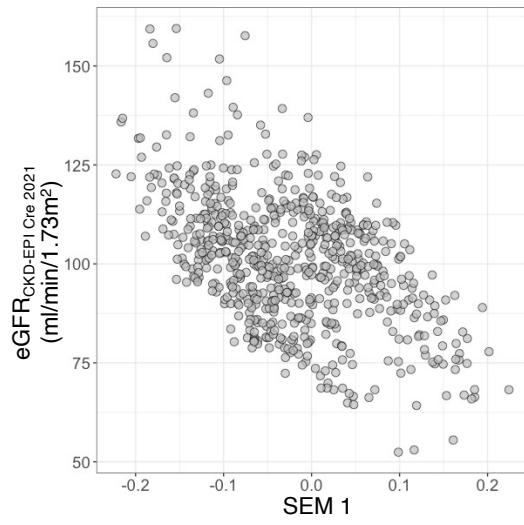**B**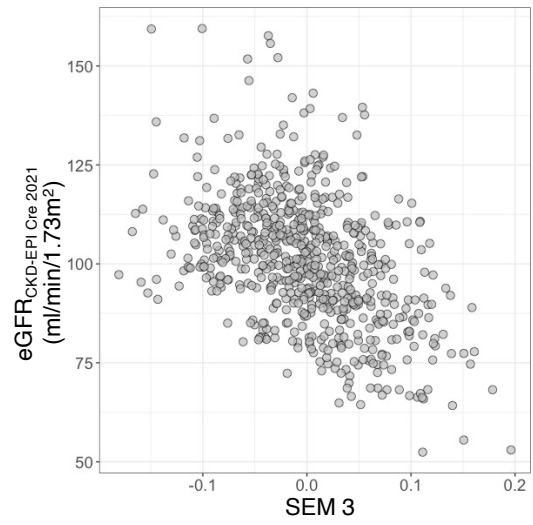**C**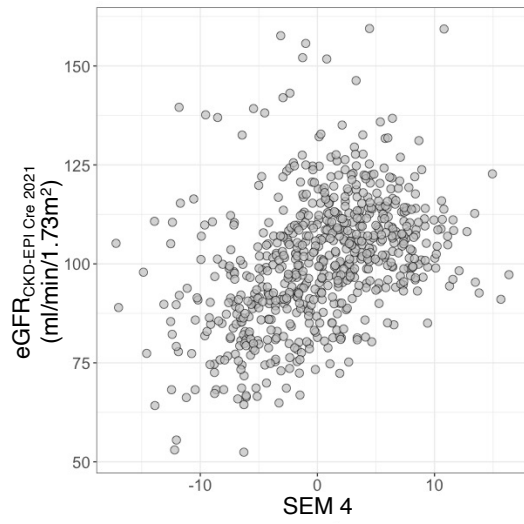

Supplement: S2 Fig — Panel A: Scatter plot of estimated with the 1st structural equation model (SEM 1) and eGFRCKD-EPI Cre 2021. Panel B: Scatter plot of estimated with the 3rd structural equation model (SEM 3) and eGFRCKD-EPI Cre 2021. Panel C: Scatter plot of estimated with the 4th structural equation model (SEM 4) and eGFRCKD-EPI Cre 2021. (PDF) [file pone.0280600.s002.pdf]

**A**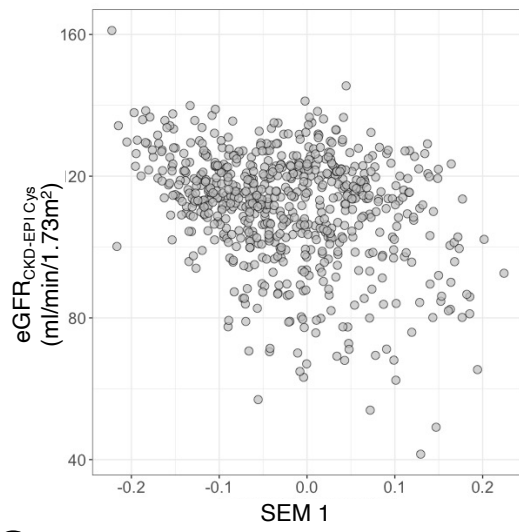**B**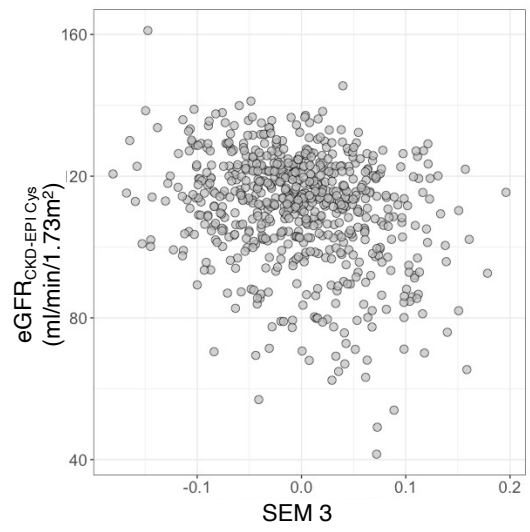**C**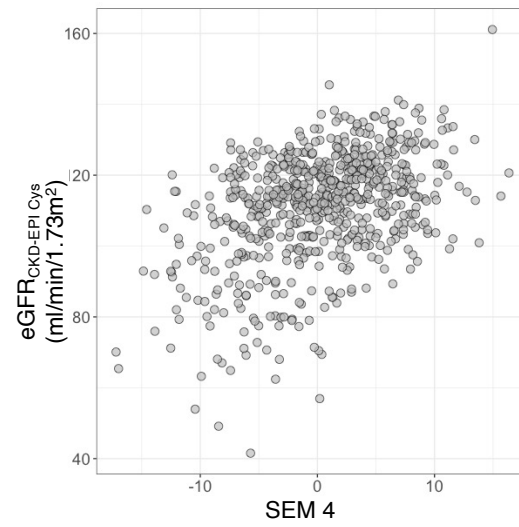

Supplement: S3 Fig — Panel A: Scatter plot of estimated with the 1st structural equation model (SEM 1) and eGFRCKD-EPI Cys. Panel B: Scatter plot of estimated with the 3rd structural equation model (SEM 3) and eGFRCKD-EPI Cys. Panel C: Scatter plot of estimated with the 4th structural equation model (SEM 4) and eGFRCKD-EPI Cys. (PDF) [file pone.0280600.s003.pdf]

**A**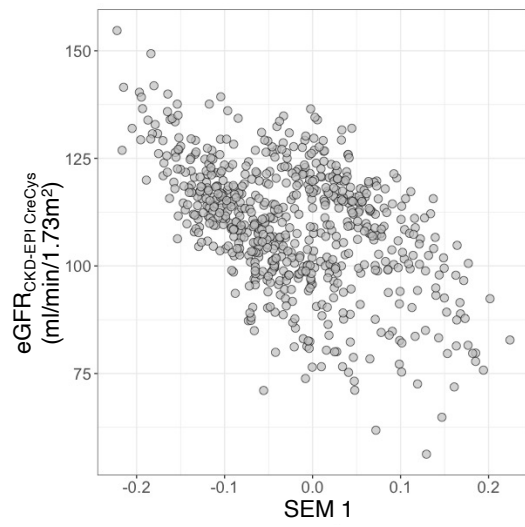**B**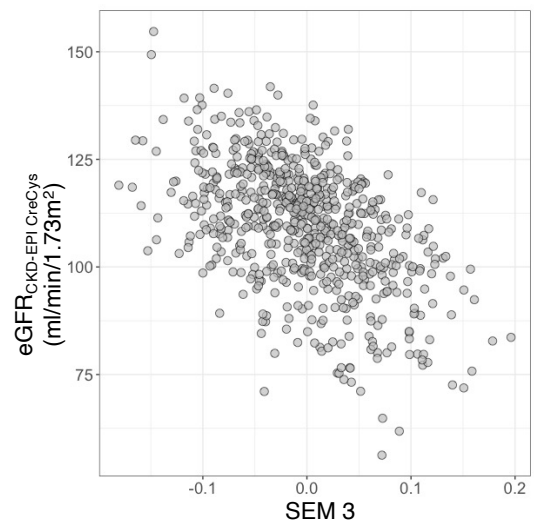**C**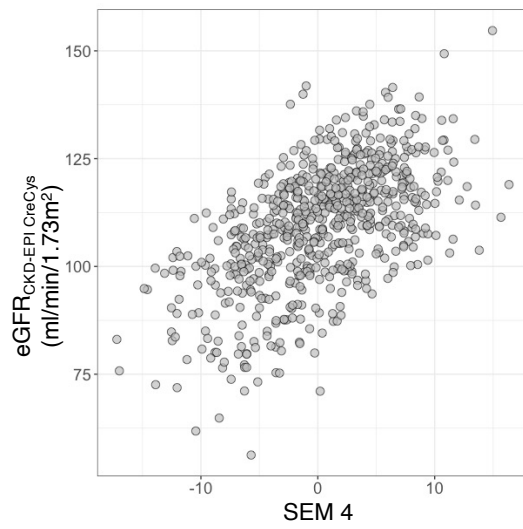

Supplement: S4 Fig — Panel A: Scatter plot of estimated with the 1st structural equation model (SEM 1) and eGFRCKD-EPI CreCys. Panel B: Scatter plot of estimated with the 3rd structural equation model (SEM 3) and eGFRCKD-EPI CreCys. Panel C: Scatter plot of estimated with the 4th structural equation model (SEM 4) and eGFRCKD-EPI CreCys. (PDF) [file pone.0280600.s004.pdf]
