# Supplementary material for: Structural equation modeling (SEM) of kidney function markers and longitudinal CVD risk assessment
Source: PLoS One. 2023 Apr 20;18(4):e0280600. doi: 10.1371/journal.pone.0280600 (PMC10118200; doi:10.1371/journal.pone.0280600)
Supplement: S1 Table — (PDF) [file pone.0280600.s005.pdf]

Supplementary Table 1. Questions, answer options, and coding for the questionnaries in the MICROS and CHRIS study

Study	Item	Question	Answer	Coding
MICROS	Age	Date of birth? -- your current age	-	continuous
	Sex	Gender	1: male<br>2: female	0: male<br>1: female
CHRIS	Age	Age at participation, rounded to whole years<br>(Date of examination - Date of birth)	-	continuous
	Sex	sex	1: male<br>2: female	0: male<br>1: female
	Race	Self-reported Non-european ancestry	0: No<br>1: Yes	0: European<br>1: Non-European
	Antihypertensive therapy	Have you ever taken medication for hypertension or high blood pressure, following a doctor's prescription?	1: Yes<br>2: No	0: No<br>1: Yes
	History of diabetes	Do you have diabetes mellitus?	1: Yes<br>2: No	0: No<br>1: Yes
	Current smoking status	Do you now smoke, as of one month ago?	1: Yes<br>2: No	0: No<br>1: Yes

CHRIS: Cooperative Health Research in South Tyrol; MICROS: three population microisolates in South Tyrol
