# Supplementary material for: Structural equation modeling (SEM) of kidney function markers and longitudinal CVD risk assessment
Source: PLoS One. 2023 Apr 20;18(4):e0280600. doi: 10.1371/journal.pone.0280600 (PMC10118200; doi:10.1371/journal.pone.0280600)
Supplement: S2 Table — (PDF) [file pone.0280600.s006.pdf]

Supplementary Table 2. Different estimation methods for GFR

Name	Formula
CKD-EPI Cre 2009	$141 * \min(SCr/k, 1)^{\alpha} * \max(SCr/k, 1)^{-1.209} * 0.993^{age} * 1.159$ [if black] * 1.018 [if female]
CKD-EPI Cre 2021	$142 * \min(SCr/k, 1)^{\alpha} * \max(SCr/k, 1)^{-1.200} * 0.9938^{Age} * 1.012$ [if female]
CKD-EPI Cys	$133 * \min(Cys/0.8, 1)^{-0.499} * \max(Cys/0.8, 1)^{-1.328} * 0.996^{Age} * 0.932$ [if female]
CKD-EPI CreCys	$135 * \min(SCr/k, 1)^{a} * \max(SCr/k, 1)^{-0.554} * \min(Cys/0.8, 1)^{-0.323} * \max(Cys/0.8, 1)^{-0.778} * 0.9961^{age} * 0.963$ [if female]
MDRD4	$175 * SCr^{-1.154} * age^{-0.203} * 1.212$ [if black] * 0.742 [if female]
MDRD6	$161.5 * SCr^{-0.999} * age^{-0.176} * BUN^{-0.17} * Alb^{0.318} * 1.18$ [if black] * 0.762 [if female]

Alb: albumin; BUN: blood urea nitrogen; CKD-EPI: CKD Epidemiology Collaboration; Cre: creatinine; Cys: cystatin; GFR: glomerular filtration rate; MDRD: modification of diet in renal disease; SCr: serum creatinine
